# Supplementary material for: DMRforPairs: identifying Differentially Methylated Regions between unique samples using array based methylation profiles
Source: BMC Bioinformatics. 2014 May 15;15:141. doi: 10.1186/1471-2105-15-141 (PMC4046028; doi:10.1186/1471-2105-15-141)
Supplement: Additional file 2 — DMRforPairs output for the comparison of A431-MCF7 and NA17018-NA17105. Please start from the HTML files in each folder. Available via the BMC Bioinformatics website. [file 1471-2105-15-141-S2.zip › 1394847754114233_MOESM2_ESM/A431_MCF7/figures/16335.pdf]

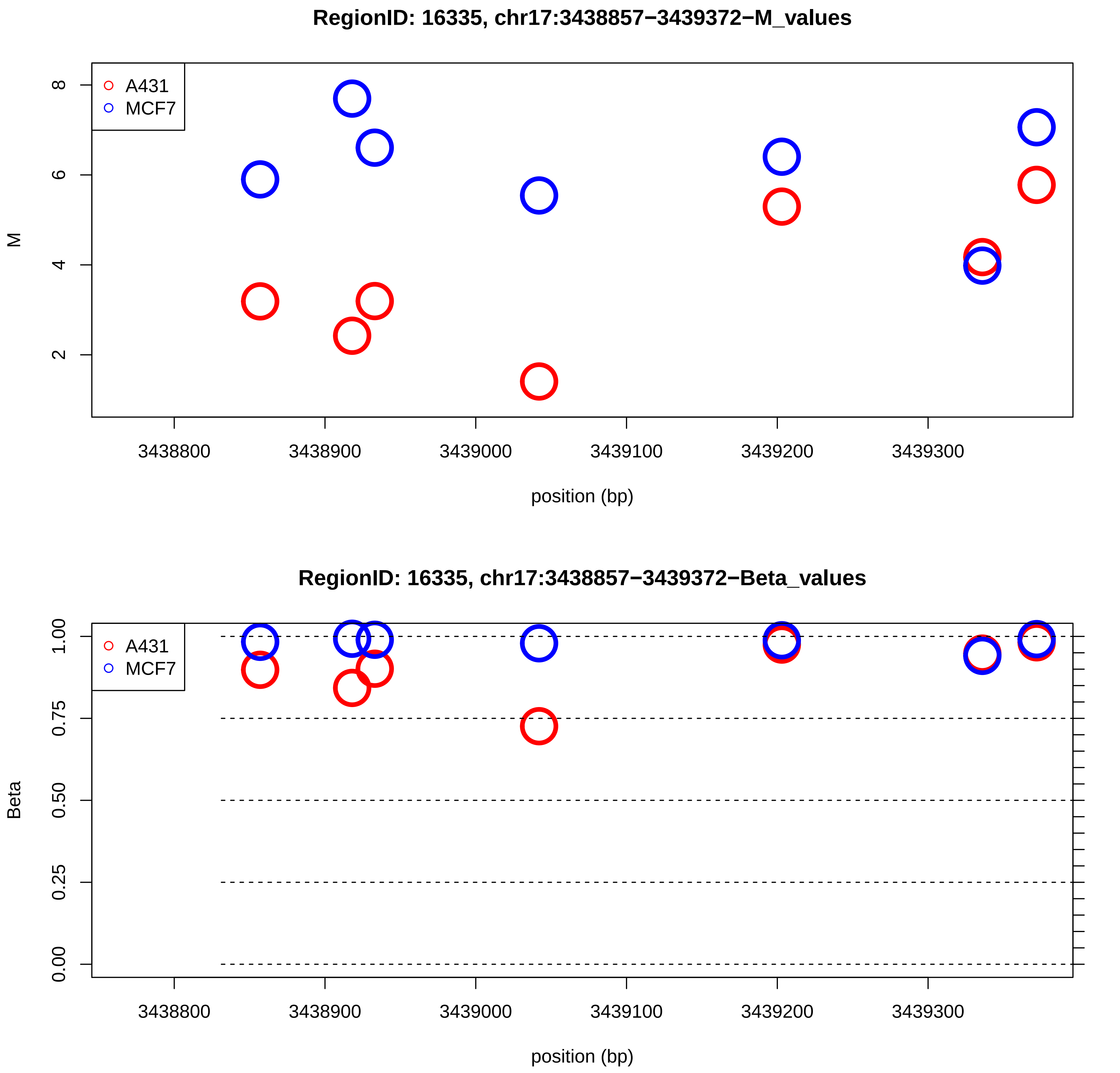
RegionID: 16335, chr17:3438857−3439372−M_values
A431
MCF7
M
2
4
6
8
3438800
3438900
3439000
3439100
3439200
3439300
position (bp)
RegionID: 16335, chr17:3438857−3439372−Beta_values
A431
MCF7
Beta
0.00
0.25
0.50
0.75
1.00
3438800
3438900
3439000
3439100
3439200
3439300
position (bp)

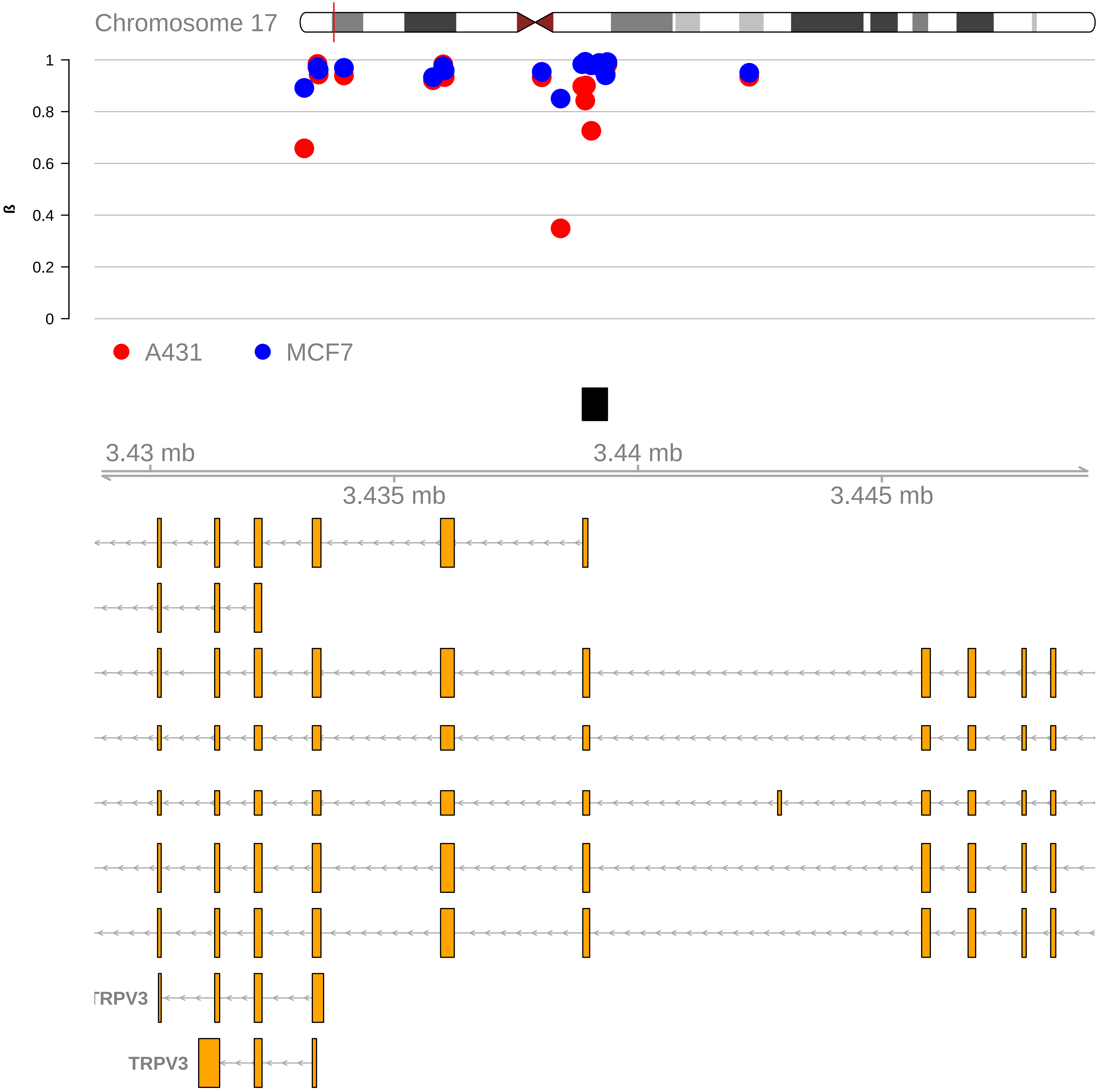

Chromosome 17
1
0.8
0.6
0.4
0.2
0
β
A431
MCF7
3.43 mb
3.44 mb
3.435 mb
3.445 mb
TRPV3
TRPV3
